# Fresh Frozen Plasma Increases Hemorrhage in Blunt Traumatic Brain Injury and Uncontrolled Hemorrhagic Shock

**DOI:** 10.5041/RMMJ.10489

**Published:** 2023-01-29

**Authors:** Hilla Abergel, Miri Bidder, Itamar Ashkenazi, Leonid Reytman, Ricardo Alfici, Michael M. Krausz

**Affiliations:** 1 Surgical Research Laboratory, Hillel Yaffe Medical Center, Hadera, Israel; 2 Technion–Israel Institute of Technology, Haifa, Israel; 3 Department of Anesthesiology, Hillel Yaffe Medical Center, Hadera, Israel; 4 Clinical Professor Emeritus, Department of General Surgery, Hillel Yaffe Medical Center, Hadera, Israel; 5 Professor Emeritus, Department of General Surgery, Hillel Yaffe Medical Center, Hadera, Israel

**Keywords:** Blood pressure, fresh frozen plasma, resuscitation, traumatic brain injury, uncontrolled hemorrhagic shock

## Abstract

**Background:**

Blunt traumatic brain injury (bTBI) and uncontrolled hemorrhagic shock (UCHS) are common causes of mortality in polytrauma. We studied the influence of fresh frozen plasma (FFP) resuscitation in a rat model with both bTBI and UCHS before achieving hemorrhage control.

**Methods:**

The bTBI was induced by an external weight drop (200 g) onto the bare skull of anesthetized male Lewis (Lew/SdNHsd) rats; UCHS was induced by resection of two-thirds of the rats’ tails. Fifteen minutes following trauma, bTBI+UCHS rats underwent resuscitation with FFP or lactated Ringer’s solution (LR). Eight groups were evaluated: (1) Sham; (2) bTBI; (3) UCHS; (4) UCHS+FFP; (5) UCHS+LR; (6) bTBI+UCHS; (7) bTBI+UCHS+FFP; and (8) bTBI+UCHS+LR. Bleeding volume, hematocrit, lactate, mean arterial pressure (MAP), heart rate, and mortality were measured.

**Results:**

The study included 97 rats that survived the immediate trauma. Mean blood loss up to the start of resuscitation was similar among UCHS only and bTBI+UCHS rats (*P*=0.361). Following resuscitation, bleeding was more extensive in bTBI+UCHS+FFP rats (5.2 mL, 95% confidence interval [CI] 3.7, 6.6) than in bTBI+UCHS+LR rats (2.5 mL, 95% CI 1.2, 3.8) and bTBI+UCHS rats (1.9 mL, 95% CI 0, 3.9) (*P*=0.005). Overall mortality increased if bleeding was above 4.5 mL (92.3% versus 8%; *P*<0.001). Mortality was 83.3% (10/12) in bTBI+UCHS+FFP rats, 41.7% (5/12) in bTBI+UCHS+LR rats, and 64.3% (9/14) in bTBI+UCHS rats.

**Conclusion:**

The bTBI did not exacerbate bleeding in rats undergoing UCHS. Compared to LR, FFP resuscitation was associated with a significantly increased blood loss in bTBI+UCHS rats.

## INTRODUCTION

Blunt traumatic brain injury (bTBI) and hemorrhagic shock (HS) commonly occur in polytraumatized patients.^1^ When both occur in the same patient, worse outcome should be anticipated.^2^,^3^ Hemorrhagic shock-related hypotension further damages brain tissue. Also, bTBI is associated with coagulopathy that may worsen HS.^4^

Optimal strategies of resuscitation for patients suffering from both bTBI and HS remain to be elucidated. Fresh frozen plasma (FFP) resuscitation may have numerous advantages. Plasma is an efficient intravascular fluid expander.^5^ It reduces the risk of dilutional coagulopathy. In animal models, plasma may have protective effects on the endothelium glycocalyx providing a mechanism for decreasing fluid extravasation and edema formation. Infusion of FFP in large animal models suffering from combined bTBI and HS reduces the size of the brain lesion and associated swelling.^6^

Due to its potential benefits, FFP was introduced in resuscitation protocols in combination with crystalloid solutions and other blood components.^7^ Up until recently, most of the data on FFP resuscitation in humans were retrospective. Two randomized trials on prehospital plasma resuscitation were published in recent years.^8^,^9^ In both trials, patients were recruited if they had evidence of at least one episode of hypotension and tachycardia or if they had severe hypotension any time before arrival at the trauma center. In the PAMPer trial, patients resuscitated with thawed plasma had lower 30-day mortality (23.2% versus 33.0%; *P*=0.03), while no differences were noted in the rate of adverse events. In this trial, plasma was provided together with crystalloid solutions in the study group. In the COMBAT trial, no differences in 28-day mortality were observed between those resuscitated only with plasma and those treated with normal saline 0.9% (15% versus 10%; *P*=0.37).

Due to the heterogenicity of patients and injury circumstances, animal models are still in place in order to evaluate the relative contribution of each intervention in a relatively uniform injury. However, most animal studies are limited to models using volume-controlled hemorrhagic shock.^10^–^13^ These models represent situations in which the animals are resuscitated only after the source of bleeding has been controlled.^10^

Our objective was to evaluate the influence of early FFP resuscitation in an animal model suffering from both bTBI and uncontrolled HS (UCHS). Unlike the controlled hemorrhagic shock model described above, UCHS examines the effect of resuscitation before the source of bleeding has been controlled.^14^–^16^ The positive effects of FFP in maintaining perfusion to the brain might be offset in part by increased bleeding caused by the “pop-the-clot” phenomenon.^17^ We therefore hypothesized that FFP will increase hemorrhage in a UCHS animal model. However, based on recently published randomized studies, we did not assume FFP resuscitation will increase mortality, leading us to adopt the null hypothesis where survival is concerned.

## MATERIALS AND METHODS

### Ethical Considerations

All experimental procedures were approved by the Institutional Animal Care and Use Ethics Committee (Technion–Israel Institute of Technology). The research protocol adhered to the National Institutes of Health Guide for Care and Use of Laboratory Animals. It was approved by the Inspection Committee on the Constitution of the Animal Experimentation (Technion–Israel Institute of Technology). All efforts were made to minimize the number of animals used and their suffering. The Arrive Guidelines were followed.^18^

### Animals

This study utilized adult male Lewis rats weighing 300–350 g (Envigo RMS (Israel), Ltd, Jerusalem, Israel). Rats were housed at the satellite animal facility at a Surgical Research Laboratory, under the supervision of the Ruth and Bruce Rappaport Faculty of Medicine of the Technion–Israel Institute of Technology. We chose male rats in order to maintain homogeneity as far as possible in a rat model in which bleeding is the main outcome evaluated. Rats were maintained at an ambient temperature of 25±1°C, with controlled humidity levels of 60% and a 12-hour light–dark cycle (from 7 a.m. to 7 p.m.). All rats had access to standard laboratory food and water ad libitum. Rats were acclimatized for 1 week before experimentation.

Rats were randomly allocated into groups according to the mechanism of trauma (without trauma, bTBI, UCHS, or bTBI+UCHS) and resuscitation protocol (without resuscitation, FFP, or lactated Ringer’s solution [LR]). Overall, there were eight groups: (1) Sham-operated; (2) bTBI; (3) UCHS; (4) UCHS+LR; (5) UCHS+FFP; (6) bTBI+UCHS, (7) bTBI+UCHS+LR; and (8) bTBI+UCHS+FFP.

No previous data were available as to the expected amount of bleeding in rats undergoing combined bTBI and UCHS. Assuming a standard deviation of 1 mL hemorrhage in rats undergoing bTBI and UCHS with no resuscitation, a sample size of 10 rats in each group had 80% power to detect a difference between means of 1.33 mL with a significance level (alpha) of 0.05 (two-tailed).

### Experimental Setting and Design

**Figure 1:**
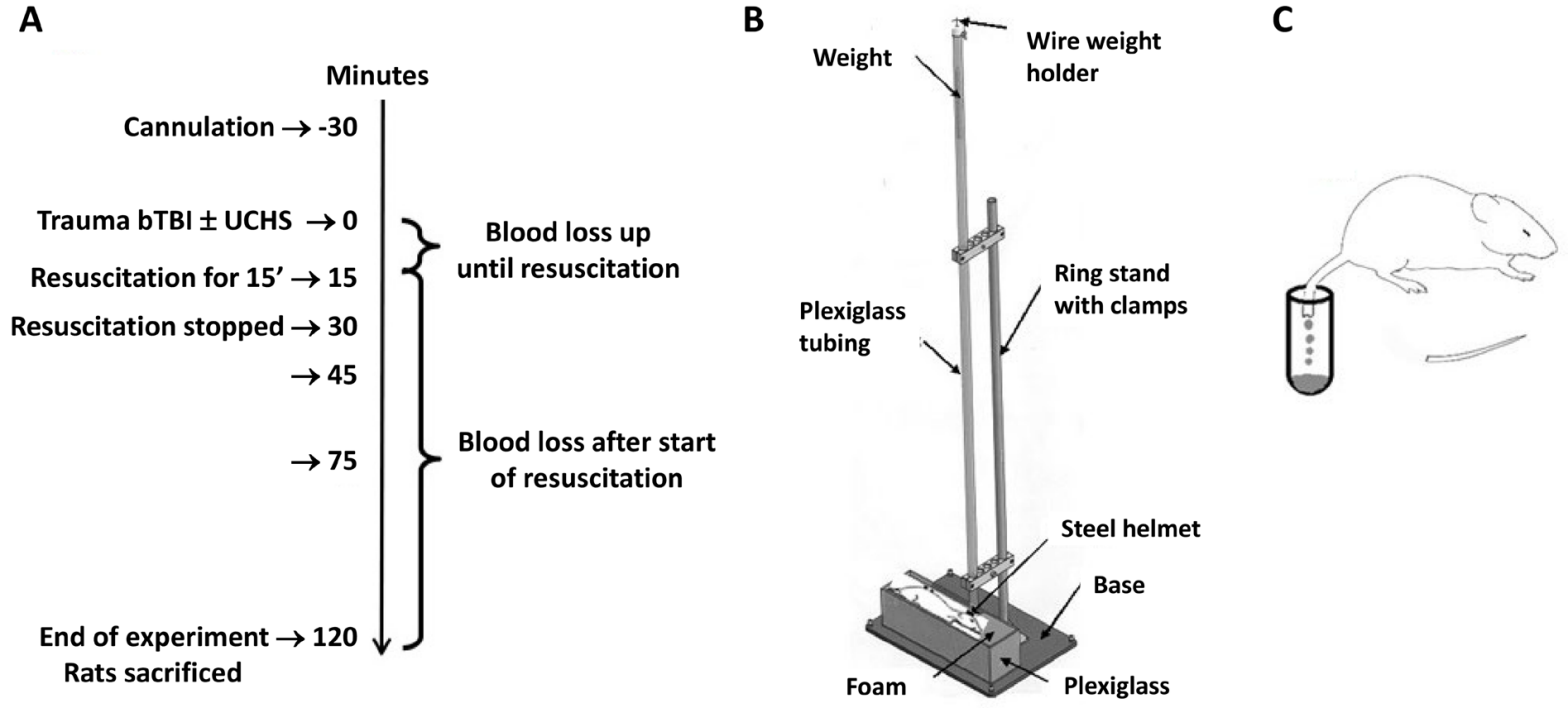
Schematic Representation of the Experiment Timeline (A) and Trauma Mechanisms: Blunt Traumatic Brain Injury (B) and Uncontrolled Hemorrhagic Shock (C).

Rats were anesthetized with pental (50 mg/kg) and fentanyl (0.65 μg/kg) in saline solution administered intraperitoneally. The right and left femoral arteries and left femoral vein were cannulated using polyethylene tubing. The right arterial line was connected to a calibrated pressure transducer and to a controlled data acquisition system (Transducer MLT0699, Amplifier ML224, PowerLab 4/30 ML866; ADInstruments, Sydney, Australia) for blood pressure monitoring. The left femoral artery was employed for blood withdrawal and the vein for intravenous fluid infusion. The rats were placed on a heating pad and rectal temperatures were monitored throughout the experiment and maintained at 37°C. According to the group allocation, the rats underwent bTBI, UCHS, bTBI+UCHS, or no injury (Sham-operated) at 0 min (Figure 1A, B, C). In rats undergoing bTBI+UCHS, the head injury was inflicted first, followed by the amputation of the tail. Once the full extent of trauma was inflicted, 15 min later, rats allocated to fluid resuscitation (LR or FFP) were resuscitated for an additional 15 min (between 15 min and 30 min). Bleeding was assessed in rats undergoing UCHS by collecting the blood shed from the resected tail into a polypropylene tube from the time of tail injury until the end of the experiment. Specific details on the injuries and resuscitation are provided below.

### Blunt Traumatic Brain Injury

The bTBI was induced by using the “weight drop model,” as previously described (Figure 1B).^19^–^21^ Briefly, an external weight of 200 g was dropped “free fall” from a 1 meter height onto a round steel disk that was adhered to the anesthetized rat’s skull. The impact point was 1 to 2 mm lateral to the midline of the skull’s convexity.

### Uncontrolled Hemorrhage

The UCHS was performed by resection of two-thirds of the rat’s tail (Figure 1C). The shed blood was collected and measured as we described previously.^22^ Briefly, the rat’s tail was measured and the area of resection calculated and marked; the tail of the rat was then amputated with the aid of a sharp knife. In rats undergoing combined injury (bTBI+UCHS), the tail was resected 5 minutes following the brain injury. The amputated end of the tail was then placed into a polypropylene test tube in which the blood shed from the tail was collected (Figure 1C).

### Resuscitation

Fifteen minutes following trauma, resuscitation with either LR or FFP was initiated in selected rats by using a syringe pump (NE-1000 Syringe pump; New Era Pump Systems, Inc., Farmingdale, NY, USA). In rats undergoing combined injury, the timing of resuscitation was set to 15 minutes after tail resection. Resuscitation was provided for 15 minutes. Lactated Ringer’s solution was chosen for comparison since it is the most common crystalloid solution used for prehospital resuscitation in Israeli civilian and military setups. Three milliliters of resuscitation solution were administered over 15 min (at a rate of 12 mL per hour); 3 mL represent 10%–15% of the rats’ blood volume.^23^ This volume is equivalent to the 500–750 mL initial resuscitation recommended in severely injured adults.^24^

### FFP Preparation

Male Lewis rats, apart from the experimental groups, were assigned as plasma donors. Lewis rats do not require blood grouping before transfusion.^25^ Rats were anesthetized, and their aorta was cannulated. Blood was withdrawn from the aorta into citrate phosphate dextrose-adenine (CPDA-1)-containing sterile Vacuette^®^ tubes (Greiner Bio-One GMBH, Kremsmünster, Austria). Blood was aspirated into these tubes at a volume ratio of 1:6 (CPDA-1:whole blood). The blood was centrifuged and the FFP was separated and stored at −70°C until needed.

### Data Collection

During the experiment, blood samples were collected into heparinized glass capillary tubes at the following time points: −30 min (end of cannulation), 0 min (after bTBI and UCHS), 15 min (before resuscitation has started), and 45 min, 75 min, and 120 min (end of the experiment). Blood lactate, base excess, hematocrit, and electrolyte levels were determined by GEM Premier 3000 (Instrumentation Laboratory, Lexington, KY, USA). Mean arterial pressures (MAP) were computed from the arterial tracing. Blood shed from the amputated tail was measured. Two periods of time were defined (Figure 1A). Blood loss up until resuscitation included the period between amputation of the tail and the start of resuscitation. In all rats undergoing UCHS, this period lasted 15 minutes, whether they were resuscitated with LR, FFP, or no fluids at all. Blood loss after the beginning of resuscitation included the period from the start of resuscitation until the end of the experiment at 120 minutes, regardless of the resuscitation type.

According to the ethics-approved protocol, the rats were sacrificed immediately at the end of the experiment while still anesthetized.

### Data Analysis

Rats were divided into groups according to their trauma mechanism and protocol of resuscitation. In each of these groups, means were calculated for each of the variables measured according to the point of time in which the measurement was made. Bleeding differences between bTBI+UCHS rats and UCHS rats before resuscitation and after resuscitation were evaluated by unpaired *t*-test. Differences in bleeding according to the different resuscitation protocols within these two different trauma groups were evaluated using ordinary one-way ANOVA. Decrease in MAP between baseline and 45 min were calculated between paired measurements in individual rats. Differences in MAP decrease between baseline and before the start of resuscitation, and differences in the extent of MAP decrease between trauma mechanisms were evaluated with Mann–Whitney tests. Decrease in hematocrit between baseline and 45 min was evaluated for individual rats. Hematocrit changes between rats with similar trauma mechanism but different resuscitation protocols were evaluated with the Kruskal–Wallis test. Differences in hematocrit (Hct) decrease between rats resuscitated with LR and no resuscitation were evaluated with the Mann–Whitney test. Differences in peak lactate within the bTBI+UCHS rats were evaluated with the Kruskal–Wallis test. Trends in individual rats between time points were explored in order to evaluate the validity of trends seen in the means of MAP and lactate. Mortality rates were compared with chi-square test for independence. Data were analyzed using dedicated statistical software programs (GraphPad Instat 3.06 and GraphPad Prism 6.00 versions for Windows, GraphPad Software Inc., San Diego, CA, USA). All tests were two-tailed. *P* values less than 0.05 were considered significant. Numbers, percentages, and 95% confidence interval (95% CI) values were approximated to the nearest decimal and *P* values to the nearest thousandth.

## RESULTS

Of the 127 male Lewis rats used in this study, 15 were excluded due to technical problems (cannula dislodgement and clogging). Fifteen other rats died immediately following trauma, either from brain injury (bTBI) or uncontrolled hemorrhagic shock (UCHS), or both (bTBI+UCHS). Ninety-seven rats that were considered immediate survivors and therefore potentially salvageable were included in the analysis (Table 1).

**Table 1:** Bleeding, Hematocrit Loss, and Mortality in Immediate Survivors Undergoing Combined Injury (bTBI+UCHS), Uncontrolled Hemorrhagic Shock (UCHS), or bTBI only.

Group Name	No. of Rats	Mean Blood Loss Until Resuscitation (95% CI)	Mean Blood Loss* After Start of Resuscitation (95% CI)	Hematocrit Loss* Between Baseline and 45 min (95% CI)	Mean Hematocrit at 120 min (95% CI)	Mortality Proportion (95% CI)
Sham	11	N/A	N/A	3.7 (2.1, 5.4)	39.5 (37.7, 41.3)	0.00 (0.00, 0.30)
bTBI	10	N/A	N/A	4.9 (2.2, 7.6)	38.5 (35.0, 42.1)	0.20 (0.50, 0.52)

UCHS	16	1.6 (0.4, 2.8)	1.7 (0, 4.0)	7.9 (6.8, 9.0)	35.5 (33.4, 37.6)	0.44 (0.23, 0.67)
UCHS+LR	11	1.9 (1.2, 2.7)	1.4 (0.3, 2.6)	8.3 (7.0, 9.6)	35.6 (33.2, 38.0)	0.36 (0.15, 0.65)
UCHS+FFP	11	1.7 (1.0, 2.5)	3.1 (1.1, 5.0)	10.5 (8.5, 12.4)	32.1 (29.4, 34.8)	0.36 (0.15, 0.65)

bTBI+UCHS	14	2.1 (0.7, 3.5)	1.9 (0, 3.9)	9.3 (7.6, 11.0)	38.5 (32.6, 44.4)	0.64 (0.39, 0.84)
bTBI+UCHS+LR	12	1.7 (1.3, 2.2)	2.5 (1.2, 3.8)	9.5 (7.6, 11.4)	36.4 (31.6, 41.2)	0.42 (0.19, 0.68)
bTBI+UCHS+FFP	12	2.3 (1.8, 2.7)	5.2 (3.7, 6.6)	13.5 (11.9, 15.1)	31.8 (27.8, 35.7)	0.83 (0.54, 0.97)

* Missing data in 7 rats.

bTBI, blunt traumatic brain injury; CI, confidence interval; FFP, fresh frozen plasma; LR, lactated Ringer’s solution; N/A, not applicable; UCHS, uncontrolled hemorrhagic shock.

### Bleeding

Following trauma, up until resuscitation (0–15 min), rats in the three groups with bTBI+UCHS bled on average 2.0 mL (95% CI 1.7, 2.3), similar to 1.8 mL (95% CI 1.7, 2.3) in the three groups with UCHS without bTBI (*P*=0.361). Once resuscitation was started, up until the end of the study (15–120 min), rats with bTBI+UCHS that were resuscitated with FFP bled considerably more than rats with bTBI+UCHS that were resuscitated with LR or not resuscitated at all (*P*=0.005) (Table 1).

Several other comparisons were evaluated. Bleeding volumes in UCHS rats were compared between subgroups according to their resuscitation protocol. Differences observed between UCHS rats resuscitated with FFP, LR, or not resuscitated at all were non-significant (*P*=0.254). Bleeding volumes in bTBI+UCHS rats resuscitated with FFP were compared to UCHS rats resuscitated with FFP. Again, the differences observed did not reach significance (*P* =0.067).

### Mean Arterial Pressures

**Figure 2:**
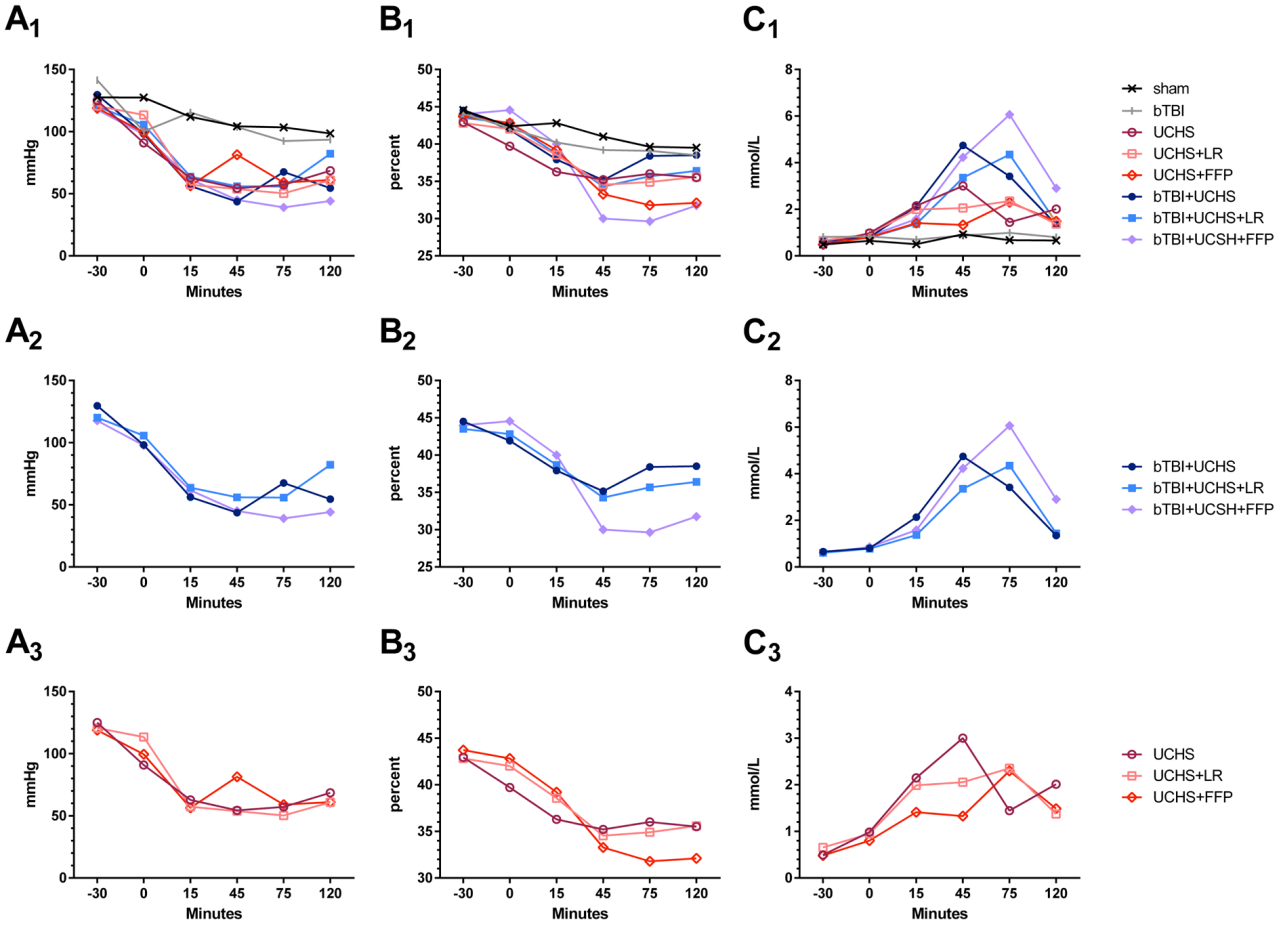
Changes in Hemodynamic Indices: Mean Arterial Pressures (A); Hematocrit (B); and Lactate Concentrations (C). A_1_-C_1_ graphs depict all groups, A_2_-C_2_ graphs depict only the groups undergoing combined injury (bTBI+UCHS), and A_3_-C_3_ graphs depict only the groups undergoing uncontrolled hemorrhage (UCHS). bTBI, blunt traumatic brain injury; FFP, fresh frozen plasma; LR, lactated Ringer’s solution; UCHS, uncontrolled hemorrhagic shock.

Following trauma at 0 min, six groups who bled showed a decrease in MAP of 40.4 mmHg (95% CI 32.2, 48.7) (*P*<0.001) (Figure 2A). No differences in MAP drop were noticed between the three groups undergoing bTBI+UCHS and the three groups undergoing UCHS without bTBI (*P*=0.679).

Following start of resuscitation at 15 min, MAP changes were variable between groups and within each group. Evaluation of individual rats within the different resuscitation groups reveals that the most common reason for increases in MAP were factitious, resulting from the early deaths of agonal animals. In this study, increases in MAP were not associated with increased bleeding. Rats with significant bleeding had low MAP throughout.

### Hematocrit

Following trauma at 0 min, Hct decreased in all six groups with uncontrolled hemorrhage, whether UCHS or bTBI+UCHS (Figure 2B). Following the start of resuscitation at 15 min, Hct levels decreased further. Only after 45 min did Hct levels stabilize. We compared rats with hematocrit levels measured at baseline and at 45 min (Table 1). In rats with combined injury bTBI+UCHS, the group resuscitated with FFP revealed the largest decrease in Hct (*P*=0.002). Similar differences were noted in rats resuscitated by FFP in the UCHS group (*P*=0.031). No differences in the extent of Hct decrease were noted when rats with bTBI+UCHS resuscitated with LR were compared to rats with similar injuries not resuscitated at all (*P*=0.881). Similarly, no differences in the extent of Hct decrease were noted between rats with UCHS resuscitated with LR and rats with a similar injury not resuscitated at all (*P*=0.594).

At the end of the experiment, hematocrit levels seemed to be the lowest for rats that underwent FFP resuscitation in either the bTBI+UCHS groups or the UCHS groups (Table 1). Indeed, final Hct levels were lower for rats resuscitated with FFP in bTBI+ UCHS groups (*P*=0.037). Lower Hct in UCHS rats resuscitated with FFP did not reach significance (*P*=0.076). It must be taken into account, however, that many animals died before the end of the experiment and this might have affected the final mean value of Hct measured in the different groups.

### Lactate

Increase in lactate levels was most noticeable in rats undergoing bTBI+UCHS (Figure 2C). In these rats, increase in lactate levels parallels the amount of bleeding noticed between groups up until resuscitation and after the start of resuscitation (Table 1). Although the peak of mean lactate levels at 75 min was higher in rats resuscitated with FFP, this difference did not reach significance (*P*=0.295).

Mean lactate levels decreased towards the end of the experiment. Evaluation of individual rats revealed that this decrease was factitious, being caused mainly by elimination of rats dying in which the lactate level measured before death was very high.

### Sodium and Potassium

**Figure 3:**
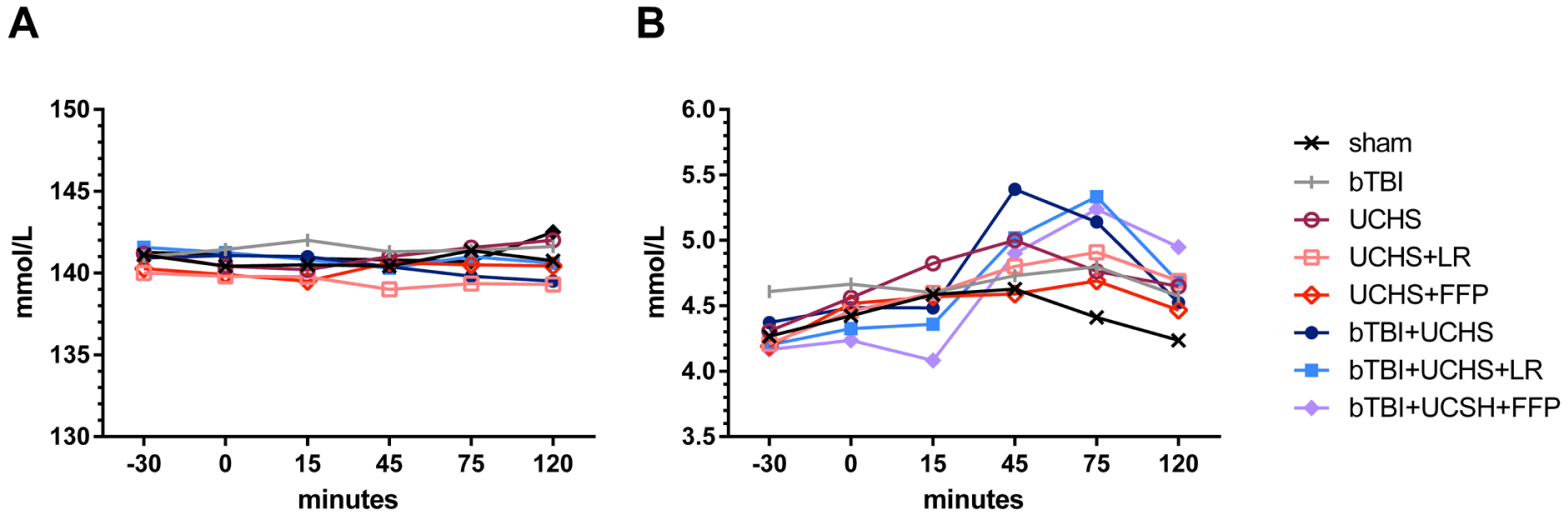
Changes in Sodium (A) and Potassium (B) Concentrations. bTBI, blunt traumatic brain injury; FFP, fresh frozen plasma; LR, lactated Ringer’s solution; UCHS, uncontrolled hemorrhagic shock.

Sodium levels remained normal throughout the experiment (Figure 3A). A slight increase in potassium levels was observed in all the rats during the observation period (Figure 3B).

### Mortality

Regardless of resuscitation protocol mortality in all three subgroups injured by bTBI+UCHS was 63.2%, while mortality in the UCHS alone groups was 39.5% (*P*=0.066). Mortality for each different group is shown in Table 1. No impact of resuscitation upon mortality was noted in rats with UCHS (*P*=0.900). Increased mortality observed in bTBI+UCHS rats undergoing resuscitation with FFP was not significant when these rats were compared to rats with similar mechanism of trauma who underwent resuscitation with LR or no resuscitation at all (*P*=0.106).

The impact of bleeding upon mortality was evaluated in 76 rats, 28 of which died (missing data in 21 rats, 13 of which died). Comparison of surviving rats to non-surviving rats reveals that bleeding was more extensive in the latter group (median 1.5 mL, IQR 0, 2.7 versus median 7.1 mL, IQR 6, 8.5; *P*<0.001). In this experiment, if bleeding was 4.5 mL or less, the risk of mortality was 8% (4/50 rats). If bleeding was above 4.5 mL, the risk of mortality increased to 92.3% (24/26 rats; *P*<0.001).

## DISCUSSION

Increased mortality observed in bTBI+UCHS rats resuscitated with FFP did not reach significance when compared to rats with the same mechanism of trauma that were resuscitated with LR or not resuscitated at all. We caution against interpreting the non-statistical significance of mortality between the bTBI+UCHS rats as signifying FFP resuscitation has no adverse effect. In this study, FFP resuscitation was associated with significant increases in bleeding in the bTBI+UCHS group of rats. Increased bleeding was associated with mortality. This has been observed in polytrauma patients with combined bTBI as well.^26^ Due to increased bleeding, the decrease in Hct levels was most pronounced in the bTBI+UCHS rats resuscitated with FFP. Though differences in levels between groups did not reach statistical significance, the lowest mean MAP levels and the highest mean lactate levels were measured in bTBI+UCHS rats resuscitated with FFP.

The extent of bleeding was higher in animals resuscitated with FFP, regardless of the injury mechanism. Though differences in bleeding volume were non-significant in the UCHS group of rats, higher volumes of blood loss suggest FFP has a detrimental effect on this model. Theoretically, FFP better conserves effective blood volume compared to LR, which may suggest that increased bleeding was secondary to “pop-the-clot” phenomena.^17^ Nevertheless, we question whether this was the underlying mechanism in our model since animals who bled the most had low blood pressures measured throughout the experiment. Lower blood pressures observed following injury throughout the experiment in bTBI+UCHS rats resuscitated with FFP suggests that low blood pressure may actually be a marker of ongoing bleeding, rather than the result of bleeding.^27^

The role of FFP in exacerbating bleeding through mechanisms other than increased hydrostatic pressures at the site of the injury is unclear. Human studies favor early and aggressive plasma resuscitation in patients with uncontrolled hemorrhage in order to prevent coagulation disorders.^28^ Still, plasma resuscitation may be associated with dilution of platelets.^29^ Having said that, LR resuscitation is associated with dilution of coagulation factors.^30^ Thus, increased bleeding following plasma resuscitation that was identified in this model should be further investigated.

In this model, inclusion of bTBI in the injury profile did not affect the extent of bleeding observed in the rats undergoing UCHS. Until resuscitation, MAP values decreased similarly in rats undergoing UCHS and rats undergoing bTBI+UCHS. The extent of hemorrhage was not different. However, once resuscitation was initiated, increased bleeding in bTBI+UCHS rats compared to UCHS rats was noted in both groups of rats who underwent resuscitation, whether FFP or LR. Though increased bleeding was not significant, we cannot rule out that resuscitation may exacerbate bleeding if bTBI coexists with UCHS. The results of this study suggest further examination of this issue in similar studies with a larger population before ruling out the possibility that bTBI exacerbates UCHS if the victims are resuscitated.

Fresh frozen plasma is rich in sodium, and resuscitation based on FFP may lead to hypernatremia.^31^ No differences in sodium levels were noted between the different resuscitation protocols employed in this study.

## LIMITATIONS

Data concerning the extent of bleeding were not collected in 21.6% of the rats. This could have affected the analysis of bleeding between survivors and non-survivors. Since all the rats with missing data, except one, belonged to either the bTBI+UCHS or UCHS groups that did not undergo resuscitation, we can still appreciate the association of bleeding with mortality in those rats that were resuscitated.

In this study, we tried to simulate the prehospital reality in which fluids can be resuscitated once prehospital medical teams reach the polytraumatized patient. Unlike reality, a predetermined volume of fluids was resuscitated, corresponding to 500–750 mL in adult humans. We did not add more fluids in order to maintain a resuscitation endpoint such as MAP. Our protocol allowed us to evaluate the extent of bleeding following equivalent volumes of resuscitation. One cannot assume that infusing more FFP in order to keep MAP beyond a certain minimum would have diminished the extent of bleeding seen in FFP-resuscitated animals.

Certain parameters were not evaluated that could have augmented our understanding of the impact of bTBI on the outcomes. We did not assess the degree of brain injury in the rats who survived the initial bTBI, and we did not evaluate other physiological parameters that either represent the degree of bTBI or may lead to adverse outcomes. These include blood pressure variability, respiratory function, and cardiac dysfunction.^32^–^34^

## CONCLUSIONS

The objective of this animal study was to simulate the period between blunt injury resulting in bTBI and uncontrolled hemorrhagic shock, and control of hemorrhage in the operating room. In this study, bTBI did not exacerbate bleeding in rats undergoing UCHS. Fresh frozen plasma resuscitation increased bleeding in rats undergoing bTBI+UCHS. A similar trend of increased bleeding with FFP resuscitation was observed in UCHS rats. Resuscitation with FFP resulted in very high mortality rates in animals with bTBI+UCHS.

## Abbreviations

CI: confidence interval
bTBI: blunt traumatic brain injury
FFP: fresh frozen plasma
IQR: interquartile range
LR: lactated Ringer’s solution
UCHS: uncontrolled hemorrhagic shock
